# Genomic and phenotypic signatures of climate adaptation in an *Anolis* lizard

**DOI:** 10.1002/ece3.2985

**Published:** 2017-07-08

**Authors:** Ariel Rodríguez, Tia Rusciano, Rickeisha Hamilton, Leondra Holmes, Deidra Jordan, Katharina C. Wollenberg Valero

**Affiliations:** ^1^ Zoological Institute Technical University of Braunschweig Braunschweig Germany; ^2^ Department of Natural Science College of Science, Engineering and Mathematics Bethune‐Cookman University Daytona Beach FL USA; ^3^ School of Integrated Science and Humanity International Forensic Research Institute Florida International University Miami FL USA; ^4^ School of Environmental Sciences University of Hull Hull UK; ^5^ Present address: Institute of Zoology University of Veterinary Medicine Hannover Hannover Germany

**Keywords:** *Anolis cybotes*, functional genomics, population divergence, recurrence, thermal adaptation

## Abstract

Integrated knowledge on phenotype, physiology, and genomic adaptations is required to understand the effects of climate on evolution. The functional genomic basis of organismal adaptation to changes in the abiotic environment, its phenotypic consequences, and its possible convergence across vertebrates are still understudied. In this study, we use a comparative approach to verify predicted gene functions for vertebrate thermal adaptation with observed functions underlying repeated genomic adaptations in response to elevation in the lizard *Anolis cybotes*. We establish a direct link between recurrently evolved phenotypes and functional genomics of altitude‐related climate adaptation in three highland and lowland populations in the Dominican Republic. We show that across vertebrates, genes contained in this interactome are expressed within the brain, the endocrine system, and during development. These results are relevant to elucidate the effect of global climate change across vertebrates and might aid in furthering insight into gene–environment relationships under disturbances to homeostasis.

## 1 Introduction

Integrated knowledge on phenotype, physiology, and genomic adaptations is required to understand the effects of climate on the fitness of organisms (Sears & Angilletta, 2011; Seebacher & Franklin, 2012). Variation in abiotic environment such as temperature influences organismal biochemical reaction rates generating organismal adaptation across all levels of biological organization (Hochachka & Somero, 2002). Across elevational gradients, temperature is linked to partial oxygen pressure and precipitation. Recently, comparative genomic and exomic studies have recovered protein‐coding genes that diverge between phenotypes adapted to different elevations (Scheinfeldt et al., 2012; Udpa et al., 2014; Yang, Qi, & Fu, 2014), but the genes that are functionally related to such environmental adaptation may be difficult to discern from the genes that adapt to other factors of selection along these gradients (e.g., available resources). This problem can be approached through the integration of independent evidence from multiple taxa, or by examining natural replicates. In two such studies, text‐ and genome mining has been used to reveal that candidate genes for climate adaptation initially identified from diverse lineages of vertebrates such as human and fish are in close functional relationship with each other (Porcelli, Butlin, Gaston, Joly, & Snook, 2015; Wollenberg Valero et al., 2014). Wollenberg Valero et al. (2014) found a network of genes identified from over 150 publications on vertebrate temperature adaptation and stress response to be functionally more closely related than expected by chance. Porcelli et al. (2015) subsequently found evidence for convergent functions of thermal adaptation genes among all Metazoans. Predicted functions align to empirically derived biochemical evidence (e.g., for vertebrate cold and freeze tolerance, Storey & Storey, 2004; Storey, 2006; Storey & Storey, 2017). Overall, these in silico studies indicate that a functional genomic network for thermoregulation and/or thermal adaptation to the environment exists, which might be regulated via similar evolutionarily conserved pathways in different vertebrate lineages, which validates a comparative approach to understanding thermal adaptation. These predictions of a conserved set of functions that adaptively evolve in response to changing abiotic environmental parameters have not been verified in a natural setting yet.

Previous studies identified a high level of determinism in the evolution of Caribbean *Anolis* lizards (Losos, Jackman, Larson, de Queiroz, & Rodriguez‐Schettino, 1998; Mahler, Revell, Glor, & Losos, 2010; Wollenberg, Wang, Glor, & Losos, 2013). Communities of *Anolis* ecology–morphology–behavior specialists (ecomorphs) are filtered by environmental niche parameters associated with elevational gradients (Wollenberg, Veith, & Lötters, 2014) and show evidence for environmental selection of thermal performance (Kolbe, Ehrenberger, Moniz, & Angilletta, 2013; Logan, Cox, & Calsbeek, 2014).

**Figure 1:**
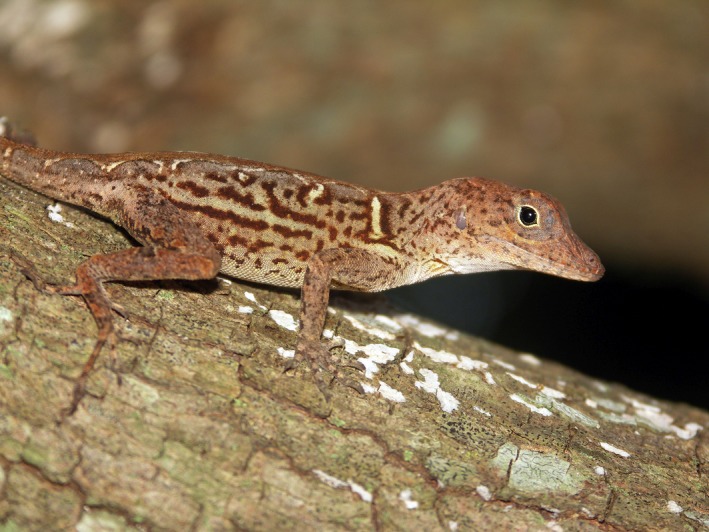
The study species, *Anolis cybotes*. Female from the Barahona peninsula on the island of Hispaniola. Copyright KCWV

The Hispaniolan species *Anolis cybotes* (Figure 1) is a common and widely distributed trunk‐ground ecomorph comprised of a cluster of genetically divergent populations including three paraphyletic taxa (*A. cybotes*,* A. armouri*, and *A. shrevei*). Intraspecific genetic divergence in this species is aligned to spatial convergence in perch use, osteomorphological measurements, toe pad scalation, physiology, and behavior (Glor, Kolbe, Powell, Larson, & Losos, 2003; Muñoz et al., 2014; Wollenberg et al., 2013). The repeated nature of these patterns across three elevational gradients inhabited by genetically divergent populations suggests that they evolved under selection, and temperature influencing physiology has been identified as a potential selective agent (Hertz & Huey, 1981; Muñoz et al., 2014; Wollenberg et al., 2013). To date, no comprehensive picture exists about the functional genomic basis of thermal adaptation and its degree of conservation across vertebrates. We hypothesize that *A. cybotes* populations inhabiting elevational gradients will exhibit signatures of genomic adaptation targeting genes, or gene functions that have previously been predicted to underlie vertebrate thermal adaptation. To test this hypothesis, we use correlative evidence to investigate environment–gene–phenotype interactions across elevational gradients.

## 2 Materials and Methods

### 2.1 RAD sequencing, genomic population structure, and outlier identification

The *A. cybotes* species complex (*A. cybotes*,* A. breslini*,* A. armouri*,* A. shrevei*) is a monophyletic clade with paraphyletic positioning of nominal taxa, in following referred to as *A. cybotes* pending taxonomic revision. It inhabits a wide range of semi‐xeric to mesic habitats from sea level to high altitudes on the island of Hispaniola (Alföldi et al., 2011; Glor et al., 2003; Wollenberg et al., 2013). Specimens of *A. cybotes* were collected from elevational gradients containing distinct populations identified in a prior publication (Glor et al., 2003), including the lowlands in the eastern portion of the islands, and highlands around the Cordillera Central, Sierra Baoruco, and Sierra de Neyba (see Table S1 for spatial distribution of samples). Genomic DNA was extracted from tail tips or muscle tissue preserved in Ethanol using a commercial extraction kit by Eurofins Genomics (Ebersberg, Germany), and DNA yield was checked with a Qubit Fluorometer. Yield was normalized to 20 ng/μl. Illumina RAD sequencing, a proven method to detect adaptive divergence across populations (Hohenlohe, Bassham, Currey, & Cresko, 2012; Hohenlohe et al., 2010), was performed by Floragenex (Oregon). Genomic DNA was digested with *Sbfl,* and each sample was barcoded with specific base tags to allow multiplexing. The resulting DNA fragments library was randomly sheared and range‐selected for fragments of 200–400 bp, to obtain an average final size of the library of 392‐bp fragments, which were then sequenced on an Illumina HiSeq 2000 using single‐end 100‐bp chemistry. We verified the extent to which *Sbfl* enzyme digestion could provide an unbiased reduced representation of an *Anolis* genome by annotating *Sbfl* restriction sites along the related species *Anolis carolinensis*’ genome using an in *silico* restriction experiment in CLC Genomics Workbench 8.0.3 (https://www.qiagenbioinformatics.com). Results indicated that this enzyme can provide a reduced representation of an *Anolis* genome (total restriction sites = 17,183) without any obvious regional bias throughout the genome (Figure S1). Quality‐trimmed Illumina reads were then aligned to a *de novo* assembled genome of *A. cybotes*. Average sample coverage was 49.11X ± 24.27 (mean:SD). SNPs with coverage >10 reads were thus genotyped from this dataset using STACKS (Catchen, Hohenlohe, Bassham, Amores, & Cresko, 2013) and later filtered using VCFtools (Danecek et al., 2011). The filtering included all SNPs genotyped in >95% of the samples and excluded those with more than two alleles and rare allele frequency <1%. Population structure was determined using three alternative methods. First, we used fastSTRUCTURE (Raj, Stephens, & Pritchard, 2014), exploring a range of *K* values (2–36) with default settings (simple prior) and later refining the search using logistic priors. Second, we used the *snmf* function in the R‐package *LEA* (Landscape and Ecological Associations, Frichot & Francois, 2015) to calculate estimates of individual admixture coefficients over a range of *K* values (1–16, parameter restrictions adjusted according to fastSTRUCTURE results). We determined the number of ancestral populations by comparing the cross‐entropy values for each *K*. Finally, we also ran principal component analyses, as implemented in LEA and the R‐package *adegenet* (Jombart & Ahmed, 2011), to visualize the variation in allelic frequencies between the samples and identify population groups.

We then used latent factor mixed models (LFMM, Frichot, Schoville, Bouchard, & Francois, 2013) to detect relationships between allele frequencies and environmental values, which is a method that takes into account and corrects for underlying population structure (Frichot et al., 2013; de Villemereuil, Frichot, Bazin, Francois, & Gaggiotti, 2014). To summarize the climatic variation across populations, we ran a principal component analysis on all Bioclim variables (bio1–bio19, from Hijmans, Cameron, Parra, Jones, & Jarvis, 2005), combined with elevation of the sampling localities and extracted five environmental principal components (Table S2). Using the first principal component that had the highest proportion of explained variance, we performed five different runs of the LFMM algorithm using 100,000 iterations and 50% burn‐in, setting the number of populations as latent factors (*k* = 6, see Section 3). We performed five different runs of the LFMM algorithm using 100,000 iterations and 50% burn‐in each. The results of the five runs were combined by calculating the median *z*‐scores and re‐adjusted *p*‐values (using the Fisher‐Stouffer procedure, according to Frichot & Francois, 2015). A list of candidate SNPs associated with the bioclimatic variable was obtained after controlling for false positives with the Benjamini–Hochberg algorithm (Benjamini & Hochberg, 1995) considering the number of tested loci (*L* = 13,652) and 0.001 false detection ratio (FDR) as implemented in *LEA* package.

Sequence reads corresponding to the significant outlier SNPs were aligned to the genome of the related green anole, *A. carolinensis* (Genome assembly AnoCar 2.0), using BLAT in ENSEMBL (V.83) and the tblastx algorithm in the software Blast2GO (Conesa et al., 2005). Fragments with significant hits in the *Anolis* genome were then searched with RepeatMasker (Smit, Hubley, & Greem, 2013) to exclude transposable elements. In summary, this analysis retained 14 protein‐coding outlier genes.

In order to visualize covariation of allele frequencies and variation in phenotype, phenotypic measurements of *A. cybotes* that are known to have produced similar phenotypic adaptations across to similar environments were obtained from the data of Wollenberg et al., 2013. These data included two variables, (1) as a measure of body condition, SVL/field weight in g, and (2) XPC1, the first principal component obtained from a PCA of osteomorphological measurements. This principal component explained 43% of osteomorphological variance and included the quantitative measurements: hindlimb metatarsal length, tibia length, fibula length, femur length, ulna length, radius length (see Wollenberg et al., 2013). The factor loadings of these original variables to the XPC1 principal component variable were negative, so results have to be interpreted accordingly. We correlated body condition values for 40 and XPC1 values of 42 of the sequenced *A. cybotes,* and frequencies of rare alleles with elevation (in STATISTICA V.13; Dell Inc.).

### 2.2 Vertebrate interactome and GO network generation

In order to infer whether newly identified protein‐coding outlier genes in *A. cybotes* fall into previously published functional categories for thermal adaptation in vertebrates (Porcelli et al., 2015; de Villemereuil et al., 2014; Wollenberg Valero et al., 2014), we constructed a functional genomic network. As protein–protein interaction data for vertebrates are most complete for humans, we used human interaction data to explore these functional categories based on the assumption of functional similarity of orthologs. Support for this approach comes from the fact that orthologs across taxa are, despite their phylogenetic definition, most commonly identified by conserved function (Altenhoff & Dessimoz, 2009). Corresponding human gene symbols of *A. carolinensis* gene symbols to which *A. cybotes* outlier loci could be mapped were subsequently used to construct a functional genomic network in CYTOSCAPE (V.3; Shannon et al., 2003). Here, we used the STRING and UniProt databases to model human gene interactions, using “human” as a taxonomy filter. The gene coding for Apolipoprotein B, *APOB*, was found as interaction partner of seven of the 14 protein‐coding outlier genes after a first trial run of the analysis and has been published to be associated with cold adaptation (Gracey et al., 2004). We therefore repeated the network with the outlier genes plus *APOB*. To infer to which extent outlier loci among *A. cybotes* populations inhabiting different elevations have similar functions as predicted for vertebrate thermal adaptation candidate genes, we then constructed a network (with Kappa 0.3) of gene ontologies and biological functions using ClueGO (V. 3.0) in CYTOSCAPE. This resulted in a list of significantly overrepresented GO terms (after term‐wide *p*‐value Bonferroni correction). We compared significantly overrepresented functions of the interactome, and functions of outlier genes, to candidate functions for thermal adaptation based on Porcelli et al. (2015), and Wollenberg Valero et al. (2014). Additionally, we annotated functions of *A. cybotes* outlier loci using information from online databases (e.g., www.genecards.org, OMIM) and literature searches. In order to explore nonrandom characteristics of the *A. cybotes* interactome, we also compared it to randomizations of itself; 100 randomizations were created using the NetworkRandomizer plugin to CYTOSCAPE (V.1.1.1., Aug. 2016). These randomizations were based on the Barabási‐Albert model, which generates scale‐free networks of a structure that is frequently encountered in nature (Albert & Barabási, 2002). The initial node degree for the algorithm was obtained from the *A. cybotes* interactome (*m* = 4). Network statistics (*t* tests) were then computed for differences between randomized and observed interactomes.

In order to test whether recovered candidate markers for thermal adaptation are represented with significantly higher proportion in the interactome than expected by chance, we also generated 50 random samples of 15 protein‐coding genes drawn from the ENSEMBL *A. carolinensis* genome annotation using the UCSC genome browser interface (accessed 9/29/2016). Interactomes and basic statistics were computed for each list of 15 markers and the number of candidate markers for thermal adaptation. *A. cybotes* outlier gene symbols were counted within these networks, and the significance of results was assessed with *t* tests.

### 2.3 Gene expression: tissue specificity in different vertebrates

The comparative functional genetic approach in our study led us to question, in which tissues the loci recovered in the lizard outlier + candidate gene network are expressed. Shared orthology of genes may predict similarity in tissue expression across vertebrate lineages. For example, Chan et al. (2009) verified strong evolutionary conservatism of tissue expression across vertebrates (pufferfish, frog, chicken, mouse, and human). On the same notion, we obtained gene expression data for outlier and candidate genes from a set of vertebrates (frog, lizard, zebrafish, chicken, and human). Expression data for human were again more exhaustive than data for other vertebrates, on which we consequently focus our interpretation. Human gene expression data measured on Affymetrix chip U133 were obtained for a list of 31 candidate genes plus 14 outlier genes from BioGPS (Wu et al., 2009) and imported into TABLEAU (V.9.0, Seattle, WA, USA) after data transformation (Isokpehi et al., 2015), and standardization to mean and standard deviation. Probe sets were compared for expression outlier values in different tissues. For this purpose, we categorized the 84 human tissue types for which gene expression is available in BioGPS into 11 tissue categories. We constructed a second expression dataset for the other vertebrates using the NCBI EST database to infer gene expression in 22 different tissue types.

## 3 Results

### 3.1 *Anolis cybotes* exhibits genomic changes in response to temperature and elevation, independently of population structure

We generated sequences from 87 samples meeting the gDNA quality threshold. Illumina Hiseq2000 sequencing was performed with a sequence threshold of 2,000,000 reads/sample, and the obtained mean number of reads per sample was 2,502,196. In total, the dataset contained 217,691,060 sequence reads. From this dataset, we genotyped 13,674 SNPs passing the reads and sample coverage filters.

**Figure 2:**
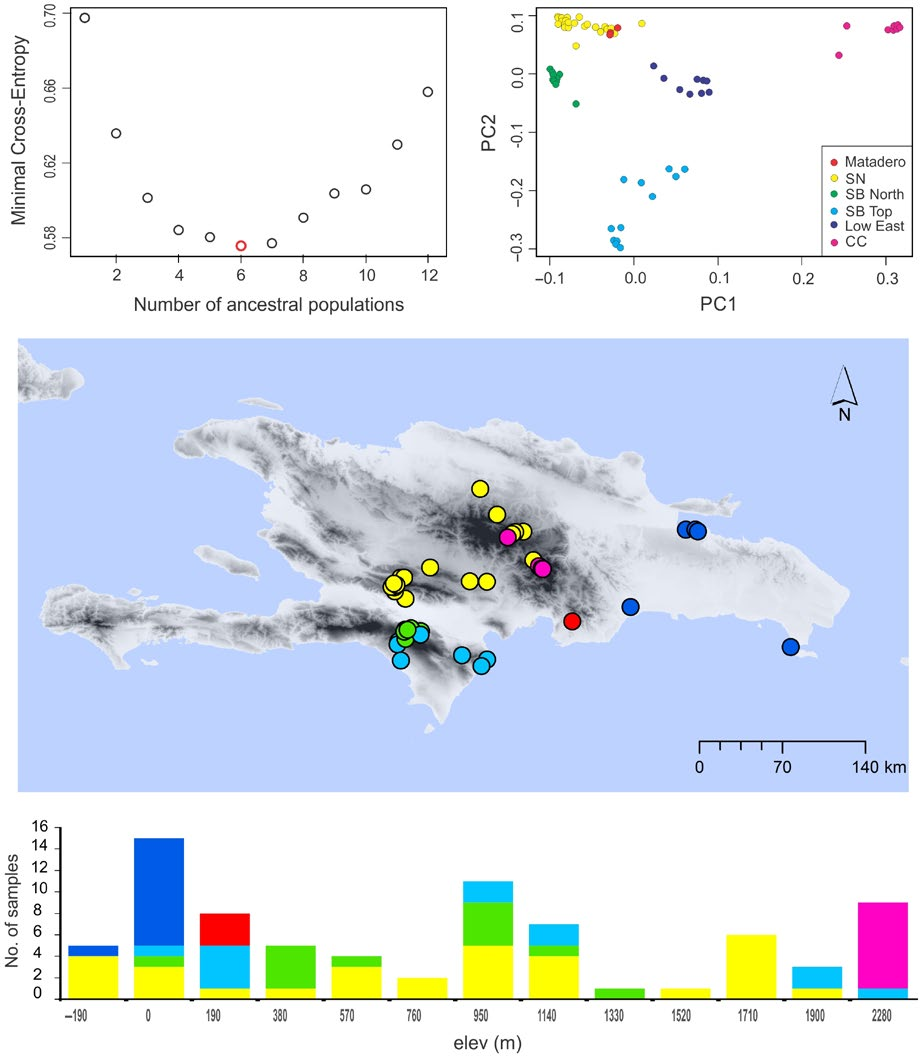
Sampling localities and genetic structure of *Anolis cybotes*. Top: Results from phylogenetic PCA and snmf‐LEA recovering six genomic populations (red—Matadero de Honduras; yellow—Sierra de Neyba; green, northern part of Sierra Baoruco; cyan—southern part and top of Sierra Baoruco; navy—eastern lowlands; pink—Cordillera Central). Center: Distribution of samples belonging to these populations across Hispaniolan mountains and lowlands. Bottom: Histogram showing the elevational distribution of the six populations and number of sequenced samples

The results of the fastSTRUCTURE runs indicated a range of *k* = 7–8 populations. However, one population had very low assignment probability values and we therefore assume *k* = 6 as the most likely number of populations of *A. cybotes* in the sample. This result was later confirmed by the cross‐entropy plots, resulting from *snmf* analysis with LEA, and the principal component analysis (Figure 2). The elevational profile shows that three mountain chains and low elevations are occupied by different populations (Figure 2).

We proceeded with the analysis with an estimate of six populations. LFMM was run on the first principal component summary variable containing elevation and temperature‐related bioclimatic variables. This PC variable contained 42% of bioclimatic variance and had significant factor loadings for elevation (negative), and (all others positive): annual mean temperature (bio1), maximal temperature of warmest month (bio 5), minimal temperature of coldest month (bio 6), mean temperature of wettest quarter (bio 8), mean temperature of driest quarter (bio 9), mean temperature of warmest (bio 10), and mean temperature of coldest quarters (bio 11, Table S2).

**Figure 3:**
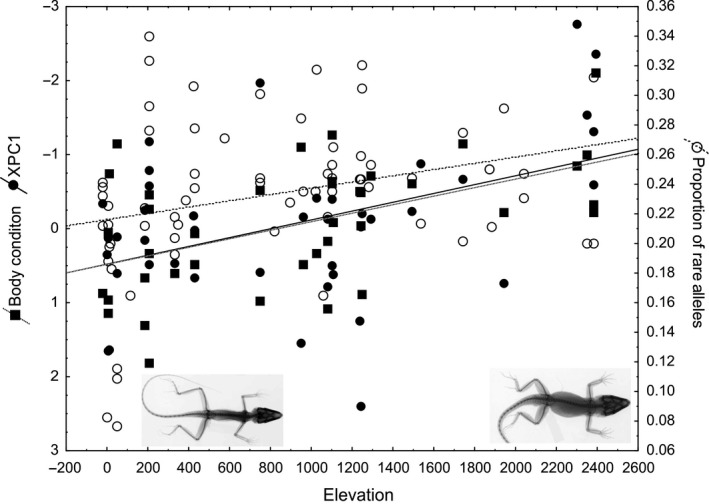
Covariation of rare allele frequencies with phenotypic adaptation to elevation. Phenotypic variables shown are published in Wollenberg et al., 2013 and represent Body condition (SVL/weight in g), and XPC1 (relative bone length, variable shows shorter bones as larger values). Inset images show X‐rays of typical lowland phenotypes of *Anolis cybotes* (left), and highland *A. cybotes* (right, own images)

The LFMM analysis recovered 84 outlier SNPs with convergent bioclimatic/elevational adaptation across the genetic populations. Of these, 33 SNPs were located within transposable elements, and 29 were either located in intergenic regions or could not be annotated. The remaining 25 SNPs were located in spliced introns, transcribed introns (evidenced by the presence of cDNA in ENSEMBL), and exons of 14 protein‐coding genes located on different chromosomes (see information provided in Table 1). Regardless of the annotation of SNPs, equal numbers of SNPs (between one and four) were contained within single RAD fragments. Of the SNPs located within the 14 protein‐coding genes, seven SNPs were located in exons of five genes, eight SNPs were located within the introns of five genes, three SNPs were located in Intron/Exon boundaries of two protein‐coding genes, and four SNPs were located in Intron/Exon boundaries of transcribed Introns (Table 1). Across all outlier SNP's, rare allele frequencies significantly increased with elevation (*r* = .287; *p* = .0186, Figure 3), as did XPC1 representing the inverse of relative bone length (*r* = .4337, *p* = .0041), and body condition (*r* = .52, *p* = .0007, Figure 3).

**Table 1:** Protein‐coding thermal adaptation loci in *Anolis cybotes* populations

RAD ID	SNP pos (Genomic location)	*z*‐Score	*P*	Human gene symbol	Human gene function *Clinical relevance*	Loc	E value annotation	ID %
54986	62 (GL344061.1:58534‐58623)	−5.26	1.9E‐7	GLTSCR2	Mitochondrial oxygen consumption^1^ *Glioma suppressor*	I	6.7E‐44	100
58260	35 (AAWZ02041818:5129‐5204)	−5.43	7.47E‐8	SIRT6	Histone modification Tumor suppressor Aging, Hypoxia	E	7.4E‐35	97
69162	39, 66 (4:134669884‐134669973)	−5.14 −4.96	3.58E‐7	PHF20	Histone modification Medulloblastoma	trI	5.1E‐39	96
87564	63; 78 (2:119357203‐119357268)	−5.12 −5.26	4.09E‐7	ASF1B	Histone Chaperone	trI	1E‐10	88
91548	89 (GL343952.1:63056‐63145)	−5.91	4E‐6	DYNC1H1*	Cell Movement CNS movement Axon Myelination^2^ *Brain malformation*	E	8.9E‐40	94
9501	34, 42, 70 (5: 132959521‐132959610)	−5.01 −4.95 −4.97	6E‐6	LRBA	Immune system *Immune deficiency w. Autoimmunity; IBS*	I	4.4E‐40	94
52394	19, 29 (2:134658466‐134658555)	−5.60 −5.12	4E‐7	MFSD6	Solute Carrier	I/E	1.8E‐35	89
10221	89 (GL343426.1:503906‐503995)	−5.91	1E‐7	MROH7/ HEATR8*	Unknown	E	2.1E‐36	90
85436	43 (GL343638.1:258914‐25900)	−5.49	5.8E‐7	RAP1GAP2	Endothelial damage repair *Asthma* ^*3*^	I	8.2E‐29	84
76510	9, 27, 40 (1:231382954‐231383015)	−4.91 −5.73 −5.41	8E‐7	RASGRP3	RAS activation *Upregulated in cancer*	I	4.2E‐24	94
72187	17 (GL343258.1:709115‐709204)	−5.06	3E‐6	SOX9	Skeletal development *Campomelic dysplasia*	I/E	3.2E‐41	96
13877	30 (GL343709.1:169329‐169348)	−5.41	8E‐7	UBE4B	Protein ubiquitination *Neuroblastoma suppressor*	I	3.8E‐03	100
27048	71 (1:263596451‐263596540)	−5.42	7E‐7	SPTBN1	Cell adhesion Bone mineral density^4^ *Cardiac Arrhythmia, Bone fracture risk*	E	2.0E‐43	99
65181	65, 77, 83 (6:21689596‐21689685)	−6.18 −5.42 −4.79	8E‐6	CALCR	Calcium excretion Ossification Osteoporosis	E	2.6E‐42	98

SNPs assigned to protein‐coding outlier loci. ID % refers to sequence similarity with *A. carolinensis* reference genome. Significance level adjusted with false‐positive discovery rate at *p* < .007. *P* = *p*‐value of outlier. Loc = SNP location: I—Intron, E—Exon, trI—transcribed Intron (RNA reads were observed via ENSEMBL.org). Clinical relevance was obtained from OMIM and PubMed, both for animal model and human. Table references (superscript numbers): (1) Yoon et al., 2014; (2) Yang et al., 2015; (3) Myers et al., 2014; (4) Estrada et al., 2012. *Not in Interactome.

### 3.2 The outlier interactome includes both predicted candidate genes and candidate functions for vertebrate thermal adaptation

**Figure 4:**
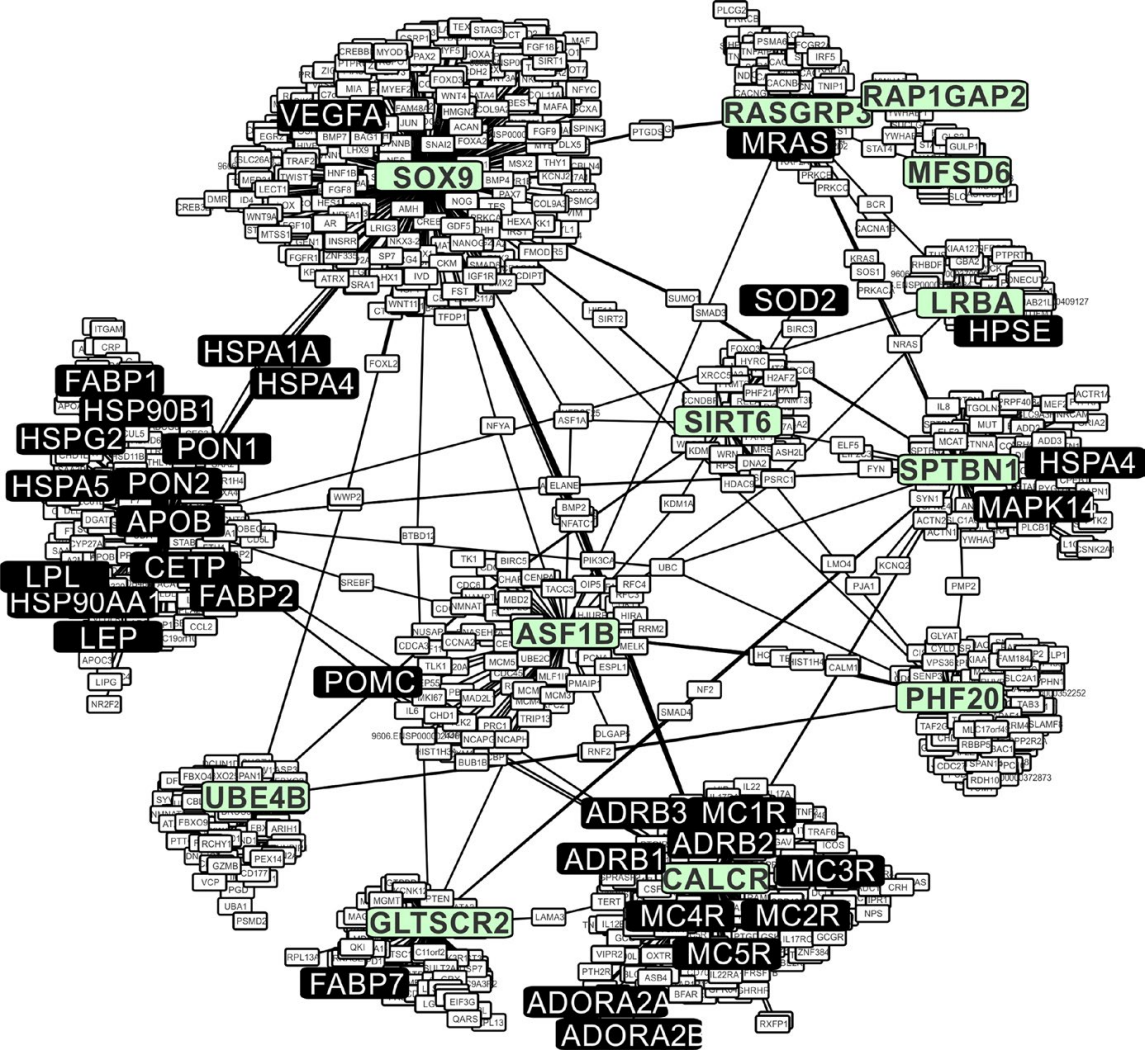
Functional genetic Interactome of thermal adaptation based on *Anolis cybotes* outlier loci. Green—*A. cybotes* outlier loci, white: Interaction partners of outlier loci. Black—Interaction partners of outlier loci that were previously predicted or evidenced to be important in vertebrate thermal genomic adaptation

We screened the network constructed in CYTOSCAPE (Figure 4) based on human orthologues of *A. cybotes* outlier loci for candidate genes previously identified in vertebrate thermal adaptation. We recovered 31 of 48 candidate genes (predicted in Wollenberg Valero et al., 2014) as direct interaction partners of the 14 protein‐coding genes identified as containing outlier SNPs in the LFMM analysis of *A. cybotes*. The interactome had 1,169 nodes and contained all outlier loci except two (*DYNC1H1* and *MROH7*, Table 1). The network had a clustering coefficient of 0.002, a network density of 0.002, a network heterogeneity of 5.806, and a closeness centrality of 0.24. These measures are informative on the information content and flow in an interactome (Ozgur, Vu, Erkan, & Radev, 2008), but have to be considered relative to other networks. In Wollenberg Valero et al. (2014), the same statistics were estimated for the 48‐candidate genes network predicted to be conserved in vertebrate thermal adaptation. Comparing parameters between these two networks reveals that the clustering coefficient constructed with *A. cybotes* outlier loci + *APOB* is lower than the predicted candidate gene network (which had a clustering coefficient of 0.02), which means that there are also discrete functional pathways contained within the *A. cybotes* outlier + candidate gene network. The network density was lower than in the original candidate gene network (which had a density of 0.005), which hints at a lower degree of common functions in the outlier + candidate gene network than in the candidate network. This is not surprising, as the RAD sequencing technique was not geared toward recovering specific genes from the original interactome and thus was more likely to reveal more distant interactions. Network heterogeneity, in contrast, was higher than in the network of 48 thermal adaptation candidate genes (3.0), which hints at a higher tendency of nodes within the *A. cybotes* outlier + candidate gene network to form hub nodes, indicating a directional flow of information. Closeness centrality was similar to the original network (0.22), which means that information speed flowing through the network is equivalent. The *A. cybotes* interactome had a significantly lower clustering coefficient (*t* = −9.9, *p* ≤ .001), closeness centrality (*t* = −40.6, *p* ≤ .001), higher centralization (*t* = 9.8, *p* ≤ .001) and higher heterogeneity (*t* = 119.7, *p* ≤ .001) than 100 randomly computed networks.

In order to test whether the presence of 17 previously predicted genes for thermal adaptation and their placement as direct interaction partners of the 14 protein‐coding outliers herein detected (empirical candidates) could have resulted by chance alone, we compared the interactome to random interactomes. We generated 50 lists of 15 protein‐coding genes randomly selected from the *Anolis* genome (UCSC genome browser, accessed 9/29/2016) and created the corresponding networks in CYTOSCAPE using identical settings. The size of these random networks varied between 41 and 3,622 genes, and to allow straightforward comparisons, the numbers of empirical candidates were expressed as relative to the number of nodes in each random network. Only in one case did the random gene lists contain one predicted gene for thermal adaptation (AQP5). The random interactomes contained between zero and five genes from both lists (at maximum, five predicted genes for thermal adaptation and one empirical candidate gene from the *A. cybotes* outlier gene list). Relative number of genes per network node for the *A. cybotes* interactome were 0.016 for the predicted genes and 0.013 for the empirical candidates. These numbers were significantly lower in the 50 random networks with an arithmetic mean of 0.0008 for the empirical candidate genes (*SD* = 0.0024, *t* = −6.47, *p* ≤ .001), and with a mean of 0.0002 for the *A. cybotes* outlier genes (*SD* = 0.0009 l, *t* = −15.38, *p* ≤ .001).

**Figure 5:**
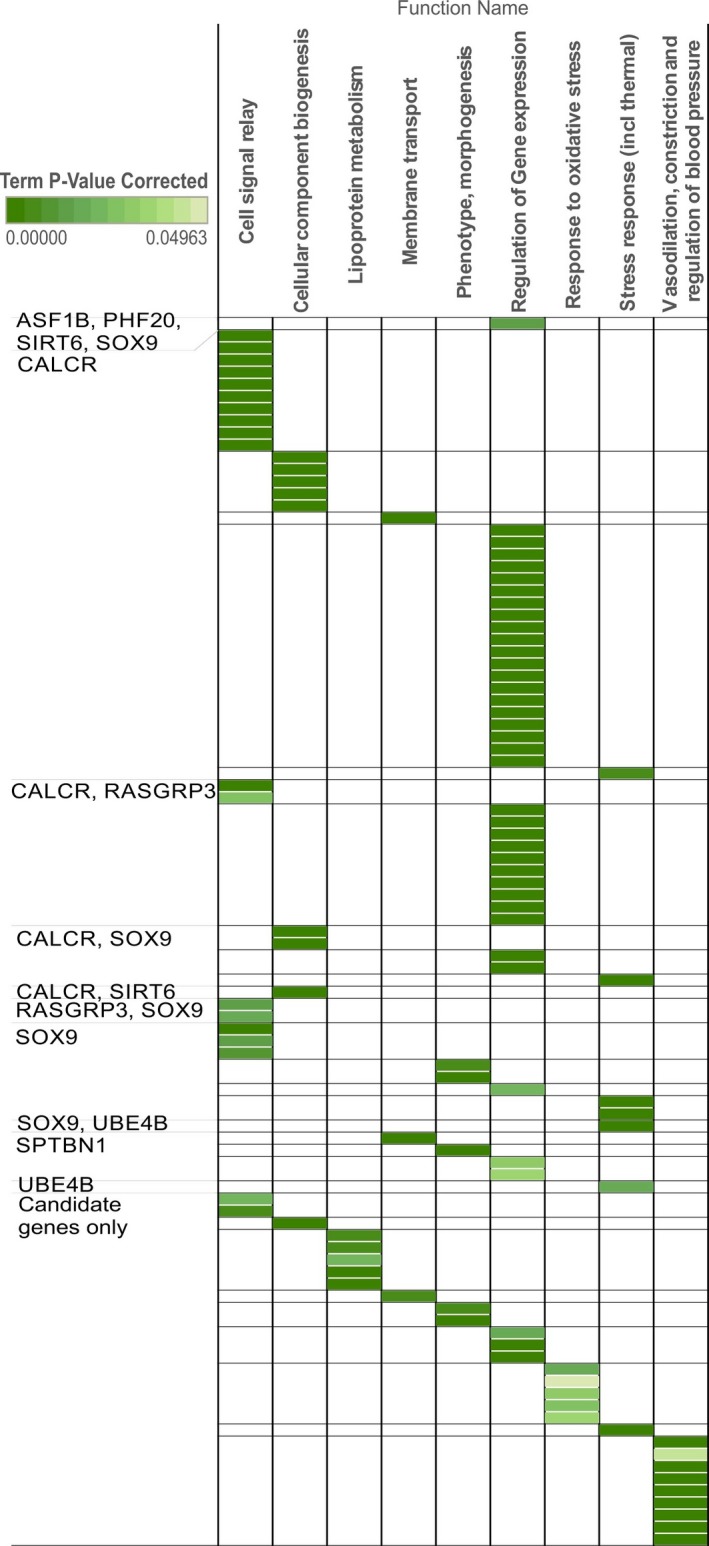
Predicted and recovered thermal adaptation functions. Matches between statistically overrepresented Gene Ontologies of *Anolis cybotes* outlier interactome presented in Figure 3 that correspond to predicted functions of climate adaptation (see Table 2). Of 120 significantly overrepresented functions in the network, 103 correspond to a predicted function for thermal adaptation. First column shows which genes or combination of genes (candidate genes only, or *A. cybotes* outlier genes) of the interactome these correspond to. Colors in table represent term *p*‐values after Bonferroni step‐down correction, complete table with GOs is available as Table S3

Functional annotation of the outlier loci (Table 1) revealed a diverse array of gene functions, with clinical significance in humans in diseases of the brain, bones, and cancer. We allocated these genes to previously predicted functions of thermal adaptation in Table 2. Functions retrieved from online resources, and the literature (given as table references in Tables 1 and 2) enabled us to match all 14 genes to at least one candidate function for thermal adaptation. Besides these single gene functions, we tested for functional similarity of the interactome containing outliers + candidate genes to previously identified candidate functions for thermal adaptation. All biological functions previously predicted to constitute the genomic basis for thermal adaptation, with the exception of muscle development and function, could be associated with significantly statistically overrepresented GOs in the interactome (Figure 5). Overrepresented GOs are informative about the common function of genes within a network, but do not necessarily represent well the functions of single genes within a network (which we have annotated in Tables 1 and 2). All candidate functions for vertebrate thermal adaptation were, as expected, significantly overexpressed by gene ontologies of candidate genes that were contained in the network as interaction partners of the lizard outlier genes. Additionally, candidate functions were also covered by outlier loci recovered from natural populations of *A. cybotes*. Most notable for their involvement in a wide range of predicted thermal adaptation functions are *CALCR* (Calcitonin receptor) and *SOX9* (Sex determining region Y box 9, Figure 5). While *SOX9* and *CALCR* are mainly involved in developmental skeletal morphogenesis, they and one other candidate gene (*SOD2*) also have a function of aging and DNA repair, which was not a predicted function for thermal adaptation.

**Table 2:** Recovered versus predicted functions of climate adaptation

A Predicted functions of thermal adaptation genes	B Candidate genes associated with predicted functions	C Outlier loci from *A. cybotes*; and their interactants
1 Lipoprotein/lipid metabolism^1,2^, Lipid transport^2^, Body mass	LPL^1^, CD36^1,6,7,8^, CETP^1^, MAPK1^1^, MAPK14^1^, SOD1^1^, STUB1^1^, LEPR^1^, FABP3^8^, UCP3^1^	CETP, LPL, MAPK14, (LEP), (APOB), **SOX9, CALCR, MROH7**, (FABP7), (FABP1), FABP2,(LPA/APOA)
2 Solute carrier Membrane channels controlling water loss/retention or cryoprotectant^1^	LPL^1^, CETP^1^	LPL, CETP, **MFSD6**
3 Stress response and homeostasis^1,2^ Incl. heat stress^1^	Heat shock proteins; HSP70/HSPA1A^1,9,10^, HSPA4^1^, HSPA5^1^, PON1^1,7^, MAPK1^1^, UCP3^1^, HSF1^1^, MAPK14^1^, HSP47^1^, UNG^1^, HSPB2^1^, SOD1^1^, STUB1^1^, HSPA8^1^	MAPK14, **GLTSCR2**, HSPA8, (HSP90AA1); HSP70/HSPA1A, HSPA4 HSPA5, PON1, (PON2) **SOX9, UBE4B**
4 Phenotypic or Phenological changes related to thermal adaptation^1^, Developmental Process^2^	Not overrepresented^1^	MC1R, MC2R, MC3R, MC4R, MC5R, POMC, **CALCR**,** SOX9**, (ADORA2A), **SPTBN1** ^11^
5 Cell signal relay^1^	MAPK1^1^, UCP3^1^, HSF1^1^, ADORA2B^1^, ADRB2^1^, MAPK14^1^, HSP47^1^, UNG^1^, HSPB2^1^, MC4R^1^, SOD1^1^, STUB1^1^, HSP48^1^, EGFR^1^, CD36^1,6,7,8^, MRAS^1^, ADORA1^1,8^	ADRB2, ADORA2B, MAPK14, MRAS, MC4R, **GLTSCR2, RASGRP3, RAP1GAP2**
6 Levels of Oxygen response, Oxidative stress response, hypoxia^1,3^ Oxidation‐reduction process^2^	UCP3^1^, SOD1^1^	(FABP1), (HSP90B1), (LEP), (SOD2), (VEGFA), **GLTSCR2** ^**4**^, (HSP90AA1), **SIRT6**
7 Muscle contraction and relaxation, muscle development^1^	ADORA2B^1^, SOD1^1^, HSPB2^1^	ADORA2B, **DYNC1H1**
8 Vasodilation^1^, blood circulation^1^, blood pressure regulation^1^, endothelial function^1^	ADORA2B^1^, SOD1^1^, POMC^1^	ADORA2B, POMC, (ADRB1), **RAP1GAP2**,** RASGRP3**, (VEGFA), (HPSE), **SPTBN1**
9 Regulation of Gene Expression^2^ incl.: Chromatin modification, Transcription^2^, RNA metabolic process^2^, RNA processing^2^, Translational Regulation, regulation of elongation^2^, Protein folding^2^, Proteolysis involved in cellular protein catabolic process^2^	No genes predicted^**1**^	**ASF1B, SIRT6, PHF20, UBE4B, SOX9**
10 Cellular component biogenesis^2^	No genes predicted^**1**^	ADORA2A, ADORA2B, ADRB1, ADRB2, ADRB3, **CALCR**, MC1R, MC2R, MC3R, MC4R, MC5R
**D Additional functions, not previously predicted**		
11 Aging and DNA repair	No genes predicted^**1**^, **CALCR** ^**6**^	**CALCR, SIRT6,** SOD2
12 Other, unknown		**LRBA, MFSD6, MROH7**

A—Predicted functions of genes underlying climate adaptation. B—Thermal adaptation candidate gene list. C—Genes verified in *Anolis cybotes* interactome. In bold: outlier loci; In regular: predicted candidate genes; In parentheses: novel genes with similar function as predicted genes. Table References (superscript numbers): (1) Wollenberg Valero et al., 2014; (2) Porcelli et al., 2015; (3) Yang et al., 2014; (4) Kim, Park, & Lee, 2012; (5) Chen et al., 2010; (6) Hancock et al., 2008; (7) Vermillion, Anderson, Hampton, & Andrews, 2015; (8) Holsinger, Schultz, & Hightower, 1996; (9) Sørensen, Dahlgaard, & Loeschcke, 2001; (10) Estrada et al., 2012.

### 3.3 Tissue‐specific gene expression of thermal adaptation genes across vertebrates shows common characteristics

**Figure 6:**
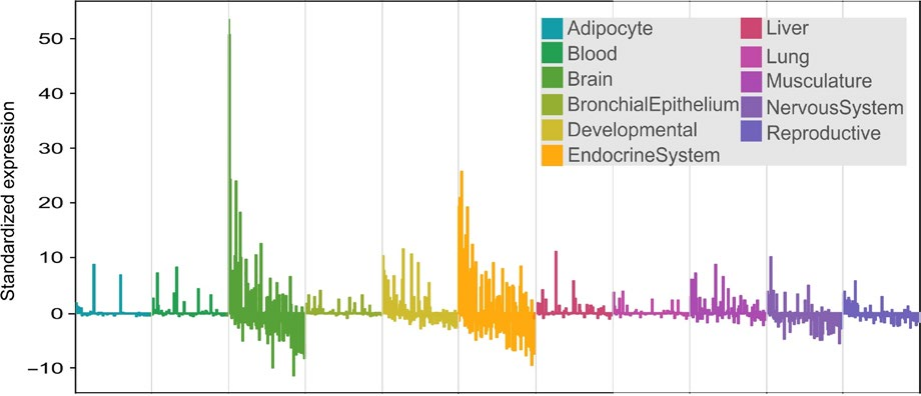
Standardized gene expression for thermal adaptation (candidate + lizard outlier) genes in humans. Categories by tissue type: Brain, Endocrine, and Developmental tissue types are the tissues showing maximal expression values across this set of genes. Color insets in bars show tissue types

**Figure 7:**
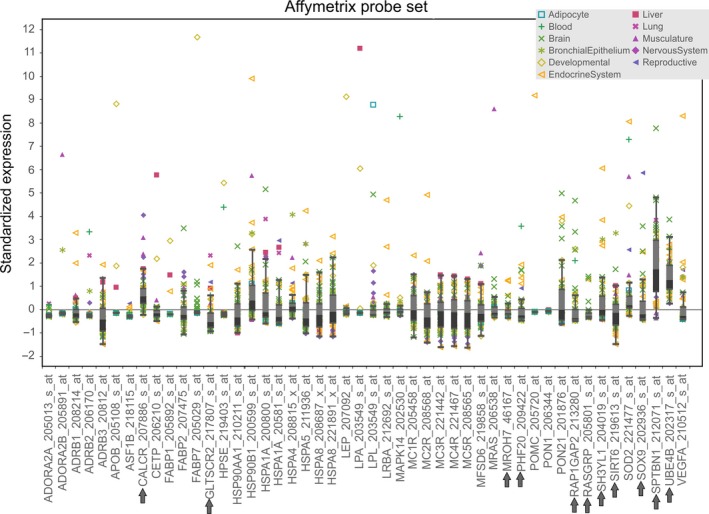
Gene‐specific expression across different tissues in human. Gray arrows denote lizard outlier loci; other genes are previously identified candidate genes for vertebrate thermal adaptation recovered from *Anolis cybotes* outlier interactome

**Figure 8:**
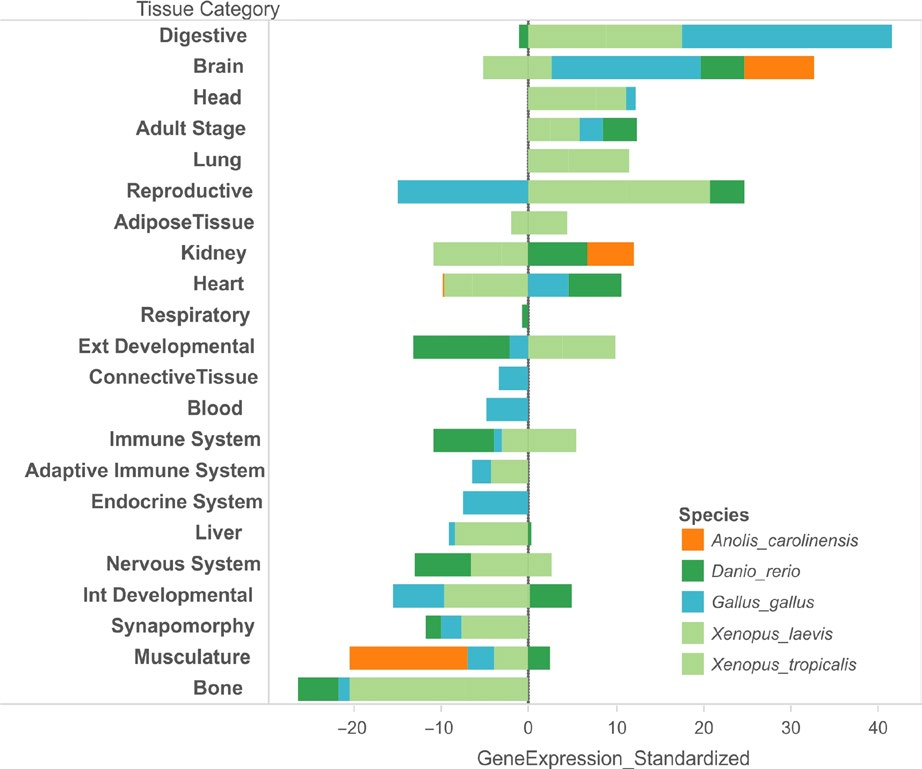
Tissue‐specific standardized expression of candidate genes in some vertebrates. Included are data from the African clawed frogs *Xenopus laevis/tropicalis*, zebrafish *Danio rerio*, chicken *Gallus gallus*, and lizard *Anolis carolinensis*. Highest expression values across these species are found in digestive tract, brain, and head. Note that tissue‐specific gene expression data are incomplete for most species. “Int and Ext Developmental” refer to internal and external “fetal” or “embryonal” tissues which, in absence of more specific information, are summarized under these categories for this manuscript. Synapomorphy—genes expressed in a structure not unambiguously comparable among species, for example, “tadpole” or “beak”

In humans, a majority of candidate + outlier genes in this climate adaptation interactome is expressed within different parts of the brain, the endocrine glands, and the developing fetus (Figure 6). Specifically, high expression values are found in the pineal gland at day and night, the pituitary gland, and in the thyroid (annotated as Endocrine system in Figure 6). Further high expression values were found in developing tissues, such as fetal brain, fetal liver, and fetal thyroid (Figure 7). Pooled gene expression data for *Xenopus laevis*,* Xenopus tropicalis*,* A. carolinensis*,* Danio rerio*, and *Gallus gallus* showed similar high expression values in the brain (Figure 8), but low expression values in bone. However, available data for tissue‐specific expression of these genes in other vertebrates are not exhaustive yet, and information for developmental stages in this database may be imprecise, so that these results need to be interpreted with some caution.

## 4 Discussion

### 4.1 Functions of *Anolis cybotes* outlier loci

Our dataset combined samples of *A. cybotes* from three montane gradients and lowlands with similar bioclimatic niches. These samples belonged to six genomic populations corresponding to clades previously identified using mtDNA (Glor et al., 2003; Wollenberg et al., 2013); 14 protein‐coding genes were found to contain outlier SNPs that could be adaptive to high elevation climates in the Dominican Republic. It is also likely that in addition to the coding genes, some of the intergenic SNPs we found to be under selection could also affect the expression of coding genes. These loci could additionally be affected by Linkage, which in turn may be functionally associated with thermal adaptation (see Hoffmann & Rieseberg, 2008). However, lacking both a properly annotated genome of the target species and a reference databases for gene regulatory element functional pathways, it is not yet possible to discern these effects yet and we therefore focus this manuscript on the functional interpretation of obtained intragenic SNPs and functions of their loci.

Several outliers were located within genes involved in bone formation and development: *SOX9*,* CALCR*, and *SPTBN1* are known to directly influence vertebrate skeletal morphogenesis, cartilage formation, and ossification (Estrada et al., 2012; Hittmeier, Grapes, Lensing, Rothschild, & Stahl, 2006). *CALCR* (the calcitonin receptor) has been associated with osteoporosis in humans (Table 1). It is also a biomarker for inflammation and stress response (Becker, Nylen, White, Muller, & Snider, 2004) and is overexpressed during retinal aging (Chen, Muckersie, Forrester, & Yu, 2010). The outlier locus *SIRT6* (Sirtuin‐6) has been identified as a biomarker for mammalian aging with DNA repair function (Mostoslavsky et al., 2006) and functions as a gatekeeper for the glucose metabolism under conditions of hypoxia (Zhong & Mostoslavsky, 2010). Locus *SOD2* (Superoxide Dismutase 2) is normally associated with the mitochondrial oxidative stress response, which has been identified as one possible factor for aging, and is a predicted function for thermal adaptation (Harman, 1956; Lund, Chu, Miller, & Heistad, 2009). Like other genes in this study, it shows high expression levels in the endocrine system, reproductive tract, brain, and during development. *SOX9* (SRY‐Box9) defects in humans can lead to skeletal malformations in early development and is involved in vertebrate tail regeneration (including *Anolis,* regulated by *TGF‐*β, Lozito & Tuan, 2015) and temperature‐dependent sex determination (Western, Harry, Graves, & Sinclair, 1999).

### 4.2 Conserved functional genetic pathways for thermal adaptation and tissue specificity

Our sequencing strategy based on RAD sequencing constitutes a reduced representation of the genome of *A. cybotes* and thus possibly could not recover all outliers for thermal adaptation in this species. The main question to be addressed in this study was however not an exhaustive genome scan, but whether outlier loci of the present dataset correspond to previously predicted functions for vertebrate thermal adaptation and are located in the same metabolic pathways. Our results unambiguously show that the adaptive response to changes in elevation‐associated climate in *A. cybotes* are mediated by the same functions as predicted for thermal adaptation across other vertebrates and study settings.

“Aging and DNA Repair” was a novel, not previously predicted function of outlier genes, and might be related to the predicted “Stress Response”/”Oxidative Stress Response” categories. The functional category “Lipid metabolism” is of known relevance for endotherm vertebrate thermogenesis, but in our study was covered by three *A. cybotes* outlier loci (*SOX9, CALCR, MROH7*). While ectotherms do not have BAT‐associated thermogenesis, these genes are also linked to hypoxia tolerance. This suggests a functional connection between lipid metabolism and hypoxia response. Hypercholesterolemia was shown to increase hypoxia tolerance in rats (Xi et al., 2006), but its link to thermal adaptation of ectotherms is not well studied and warrants further attention. Similar to human altitude adaptation (Beall, 2000; Bigham et al., 2010), the hypoxia pathway might be a target of ectotherm thermal adaptation, as low temperatures induce hypoxia (Bickler & Buck, 2007).

Independent support for our findings of conserved functional categories for reptile thermal adaptation comes from a recent study of another anole species, *A. carolinensis*, which recovered a genomic outlier locus for latitudinal climate adaptation with one of our predicted candidate functions “vasodilation, constriction and regulation of blood pressure” (Campbell‐Staton, Edwards, & Losos, 2016).

Comparisons of the predicted thermal adaptation network (from Wollenberg Valero et al., 2014) with the *A. cybotes* outlier network revealed comparable properties of information content and information flow, but less common functions of network nodes. This may be an outcome of the random nature of the RAD sequencing strategy not being targeted to capture those specific genes predicted to be adaptive to thermal selection. Network randomizations revealed that the network structure of the *A. cybotes* interactome indicates more discrete functional pathways, a lower speed of information flow, and a more centralized structure with a higher tendency to contain hub nodes than the structure of random networks drawn from the same set of genes. This result also agrees with our hypothesis that discrete functional categories mediate thermal adaptation on a genomic level. Furthermore, comparisons of the *A. cybotes* interactome with randomly generated networks indicate that the high number of previously predicted genes recovered in this network is unlikely to be a random event.

We found gene orthologs of the *A. cybotes* interactome in humans to be highly expressed within the brain and endocrine system. Like in mammals, the endocrine system of reptiles and amphibians is involved in various aspects of bio‐regulation, such as temperature, oxygen, and humidity homeostasis. Additionally, it regulates molting and metamorphosis. In reptiles, thyroid removal reduces rates of oxygen consumption (Norris & Carr, 2013). These expression patterns corroborate the newly discovered interactomes’ likely importance in vertebrate adaptation to changing environments. Similar results of high expression of the same genes in brain and head were found in other vertebrates (frog, fish, chicken, and the lizard *A. carolinensis*). A developmental role of thermal adaptation genes evidenced by their expression during early development and their functions as regulators of morphology during ontogenesis means that thermal adaptation may promote modifications during early development.

### 4.3 Relationship between genotype, phenotype, and environment

Among the predicted biological functions of outlier loci, response to temperature and stress have previously been linked to phenotypic modification of *A. cybotes*: Muñoz et al. (2014) already suggested that *A. cybotes* is under greater selective pressure to adapt to cold temperatures at high elevations than to hot temperatures at low elevations. Across *A. cybotes* populations, average frequency of rare alleles of the intragenic SNPs increased with elevation, along with increasing body mass and decreasing length of limbs. Body condition is related to body mass (Green, 2001), which may be determined by water content, bone mass and density, or adipose tissue mass. Despite the ability of this lizard species to behaviorally compensate for temperature fluctuation, we can show here that its phenotypic divergence is aligned with genomic divergence in response to elevation‐related change in climate. We suggest that the observed phenotypic variation may have a functional basis in adaptive evolution of genes involved in bone formation and adipose tissue content. Abiotic factors other than temperature, such as precipitation and relative humidity, also co‐vary with elevation. While temperature is the abiotic factor most likely shaping reptile diversity across global elevational gradients (McCain, 2010), the challenge remains to disentangle the effect of these different abiotic climate variables on organismal evolution.

The fact that *A. cybotes* shows threefold recurrent similar phenotypic and ecological adaptations in different populations across the Cordillera Central, Sierra Baoruco, and Sierra de Neyba, and that repeated genomic changes arise in genes with similar functions across this dataset, can serve as first evidence from natural populations for the in *silico* derived hypothesis that functional pathways mediating environmental adaptation across vertebrates may be conserved (Porcelli et al., 2015; Wollenberg Valero et al., 2014).

## 5 Conclusion

In conclusion, our results show that genes for vertebrate thermal adaptation can be functionally classified and seem to be conserved through evolution. While the finding of genomic in concert with phenotypic adaptation across several genetically distant populations inhabiting similar environmental gradients is indicative of recurrent adaptive responses to similar environmental selection, it does not necessarily imply convergent evolution. More research is needed to determine whether the observed pattern is an outcome of convergent evolution, of parallel evolution, or the result of recurrent selection on standing variation.

We have identified signatures of selection in several genes across three elevational gradients in a lizard. The gene functions involved suggest a connection between long‐term evolutionary adaptation to an environmental selective agent, and short‐term stress response in individual organisms, which emerges as an interesting novel perspective for further study.

## Conflict of Interest

None declared.

## Author Contributions

KWV conceived and designed the study and collected samples. AR and KWV performed the laboratory work, analyzed the data and wrote the manuscript. TR, RH, LH, DJ contributed to data analysis and wrote the manuscript. All authors approved of the final version.
