# Supplementary material for: Genomic and phenotypic signatures of climate adaptation in an Anolis lizard
Source: Ecol Evol. 2017 Jul 8;7(16):6390–403. doi: 10.1002/ece3.2985 (PMC5574798; doi:10.1002/ece3.2985)

**Supplementary Figure 1. Results of in-silico experiment *Sbfl* restriction sites across the genome of *Anolis carolinensis*.** In this plot each chromosome sequence was artificially circularized and the hash lines around the circles indicate the restriction sites of *Sbfl* enzyme. Small gaps observable in chromosomes 1-6 correspond to masked regions of the genome including highly repetitive sequences.


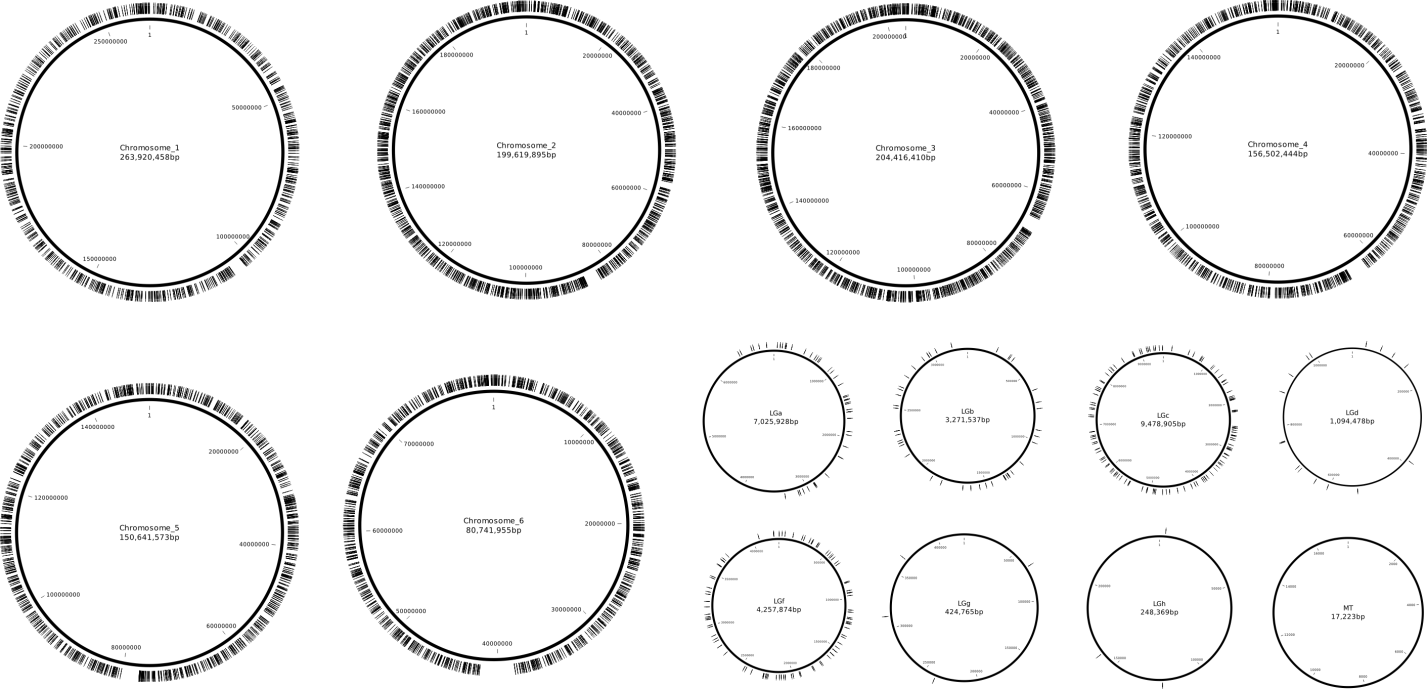

Supplement: Supplementary file 1 [file ECE3-7-6390-s001.docx]
