# Supplementary material for: Genomic and phenotypic signatures of climate adaptation in an Anolis lizard
Source: Ecol Evol. 2017 Jul 8;7(16):6390–403. doi: 10.1002/ece3.2985 (PMC5574798; doi:10.1002/ece3.2985)
Supplement: Supplementary file 2 [file ECE3-7-6390-s002.docx]

**Supplementary Table X.** Table of sampling coordinates, elevations, and genetic population structure of samples determined with adegenet.

| **Obs #** | **N (lat)** | **W (long)** | **elev(m)** | **adgenet population** |
| --- | --- | --- | --- | --- |
| **444** | 18.56191 | -71.69709 | -22 | SN |
| **445** | 18.56191 | -71.69709 | -22 | SN |
| **446** | 18.56191 | -71.69709 | -22 | SN |
| **449** | 18.56180 | -71.69652 | -19 | SN |
| **417** | 19.08695 | -69.56917 | -2 | LowEast |
| **732** | 18.1962 | -68.77321 | 2 | LowEast |
| **604** | 19.07316 | -69.47743 | 6 | LowEast |
| **605** | 19.07316 | -69.47743 | 6 | LowEast |
| **420** | 19.08660 | -69.50590 | 8 | SB_North |
| **421** | 19.08681 | -69.49753 | 13 | LowEast |
| **418** | 19.08719 | -69.56876 | 18 | LowEast |
| **428** | 18.10215 | -71.07711 | 22 | SB |
| **600** | 19.04605 | -69.43353 | 22 | LowEast |
| **415** | 19.28184 | -69.25887 | 23 | LowEast |
| **737** | 18.50012 | -69.98857 | 51 | LowEast |
| **738** | 18.50012 | -69.98857 | 51 | LowEast |
| **739** | 18.50012 | -69.98857 | 51 | LowEast |
| **450** | 18.05050 | -71.11909 | 115 | SN |
| **552** | 18.799511 | -71.509469 | 184 | SN |
| **553** | 18.799511 | -71.509469 | 184 | SN |
| **520** | 18.09324 | -71.73060 | 205 | SB |
| **521** | 18.09324 | -71.73060 | 205 | SB |
| **522** | 18.09324 | -71.73060 | 205 | SB |
| **523** | 18.09324 | -71.73060 | 205 | SB |
| **456** | 18.38984 | -70.43089 | 334 | Matadero |
| **457** | 18.38984 | -70.43089 | 334 | Matadero |
| **458** | 18.38984 | -70.43089 | 334 | SN |
| **463** | 18.39072 | -70.43110 | 349 | Matadero |
| **659** | 19.39635 | -71.12908 | 388 | SN |
| **493** | 18.31611 | -71.57899 | 423 | SB_North |
| **490** | 18.31087 | -71.58217 | 427 | SB_North |
| **491** | 18.31087 | -71.58217 | 427 | SB_North |
| **492** | 18.31087 | -71.58217 | 427 | SB_North |
| **498** | 18.33804 | -71.65513 | 575 | SB_North |
| **539** | 18.61772 | -71.77665 | 750 | SN |
| **546** | 18.61772 | -71.77665 | 750 | SN |
| **547** | 18.61772 | -71.77665 | 750 | SN |
| **551** | 18.72197 | -71.73409 | 822 | SN |
| **691** | 19.07112 | -70.8022 | 898 | SN |
| **864** | 18.32697 | -71.68158 | 950 | SB_North |
| **555** | 18.72276 | -71.70624 | 964 | SN |
| **668** | 19.20166 | -71.00102 | 1023 | SN |
| **507** | 18.21506 | -71.75635 | 1028 | SB |
| **535** | 18.13370 | -71.26699 | 1060 | SB |
| **440** | 18.32868 | -71.69934 | 1081 | SB_North |
| **442** | 18.32868 | -71.69934 | 1081 | SB_North |
| **692** | 19.06818 | -70.86378 | 1102 | SN |
| **695** | 19.06818 | -70.86378 | 1102 | SN |
| **696** | 19.06818 | -70.86378 | 1102 | SN |
| **822** | 18.25875 | -71.69658 | 1106 | SB_North |
| **905** | 18.64867 | -71.77963 | 1236 | SN |
| **906** | 18.65207 | -71.80185 | 1242 | SN |
| **907** | 18.65207 | -71.80185 | 1242 | SN |
| **505** | 18.23688 | -71.75299 | 1251 | SB |
| **506** | 18.23688 | -71.75299 | 1251 | SB |
| **703** | 19.0538 | -70.88746 | 1282 | SN |
| **823** | 18.32175 | -71.69783 | 1292 | SB_North |
| **826** | 18.31292 | -71.70493 | 1495 | SB_North |
| **711** | 18.85533 | -70.72522 | 1537 | SN |
| **909** | 18.666517 | -71.76508 | 1741 | SN |
| **910** | 18.666517 | -71.76508 | 1741 | SN |
| **911** | 18.666517 | -71.76508 | 1741 | SN |
| **917** | 18.69383 | -71.20833 | 1872 | SN |
| **921** | 18.69383 | -71.20833 | 1872 | SN |
| **914** | 18.6773 | -71.78353 | 1884 | SN |
| **916** | 18.69165 | -71.078658 | 1946 | SN |
| **443** | 18.29351 | -71.70766 | 2044 | SB |
| **500** | 18.29351 | -71.70766 | 2044 | SB |
| **712** | 18.81078 | -70.68371 | 2303 | CC |
| **719** | 18.78885 | -70.6565 | 2338 | CC |
| **833** | 18.2899 | -71.58166 | 2348 | SB |
| **764** | 18.78963 | -70.65776 | 2350 | CC |
| **769** | 18.78797 | -70.65477 | 2350 | CC |
| **704** | 19.0271 | -70.92066 | 2383 | CC |
| **705** | 19.0271 | -70.92066 | 2383 | CC |
| **716** | 18.79185 | -70.66222 | 2396 | CC |
| **760** | 18.79163 | -70.6629 | 2409 | CC |
