# Supplementary material for: Genomic and phenotypic signatures of climate adaptation in an Anolis lizard
Source: Ecol Evol. 2017 Jul 8;7(16):6390–403. doi: 10.1002/ece3.2985 (PMC5574798; doi:10.1002/ece3.2985)
Supplement: Supplementary file 3 [file ECE3-7-6390-s003.docx]

**Supplementary Table 2**. Factor loadings for Principal Component Analysis of environmental variables (elevation, and 19 bioclimatic variables extracted from WORLDCLIM). Varimax-raw rotated coordinate system, bold loadings are > 0.7. Expl. Var – Explained Variance. Prop. Totl. Var. – Proportion of total variance.

| **variables** | **PC1** | **PC2** | **PC3** | **PC4** |
| --- | --- | --- | --- | --- |
| bio1 | **0.978** | 0.063 | -0.129 | 0.142 |
| bio2 | -0.391 | -0.328 | 0.094 | -0.843 |
| bio3 | -0.030 | -0.391 | 0.689 | -0.171 |
| bio4 | -0.153 | 0.512 | -0.630 | -0.477 |
| bio5 | **0.982** | 0.042 | -0.173 | -0.010 |
| bio6 | **0.951** | 0.111 | -0.117 | 0.260 |
| bio7 | -0.417 | -0.244 | -0.090 | -0.867 |
| bio8 | **0.979** | 0.024 | -0.091 | 0.157 |
| bio9 | **0.970** | 0.080 | -0.130 | 0.158 |
| bio10 | **0.974** | 0.074 | -0.155 | 0.130 |
| bio11 | **0.979** | 0.036 | -0.101 | 0.166 |
| bio12 | -0.191 | 0.404 | **0.881** | 0.0004 |
| bio13 | -0.474 | 0.172 | **0.787** | -0.190 |
| bio14 | 0.086 | **0.921** | 0.250 | 0.202 |
| bio15 | -0.313 | **-0.819** | 0.366 | -0.084 |
| bio16 | -0.261 | 0.072 | **0.925** | -0.005 |
| bio17 | 0.062 | **0.946** | 0.250 | 0.136 |
| bio18 | -0.090 | 0.098 | 0.958 | 0.119 |
| bio19 | 0.053 | **0.966** | 0.101 | 0.113 |
| Elevation | **-0.943** | -0.114 | 0.061 | -0.187 |
| **Expl.Var** | **8.322** | **4.182** | **4.449** | **2.066** |
| **Prp.Totl** | **0.416** | **0.209** | **0.222** | **0.103** |
